# Laboratory Testing of FBGs for Pipeline Monitoring

**DOI:** 10.3390/s20133797

**Published:** 2020-07-07

**Authors:** Andrea Carlino, Alberto Godio

**Affiliations:** Department of Environment, Land and Infrastructure Engineering, Politecnico di Torino, Corso Duca degli Abruzzi 24, 10129 Torino, Italy; andrea.carlino@studenti.polito.it

**Keywords:** FBG sensors, pipelines, laboratory experiment, buckling

## Abstract

The monitoring of the effects of geohazards on pipelines can be addressed by optical fiber Bragg gratings (FBGs). They are sensitive to strain and bending, and are installed on the external surface of pipelines at discrete locations. A joint approach of theoretical analysis and laboratory experiments is useful to check the reliability of the performance of this technology. We focus on the theoretical analysis of pipeline buckling and investigate the reliability of FBG monitoring both by examining the analytical model available and by performing a laboratory-scale experiment. The novelty lies in the analysis of models and methods originally developed for the detection of pipeline upheaval buckling caused by externally imposed forces in the context of service loads (temperature). Although thermal strain is very relevant in view of its potentially disruptive effects on both pipelines and the FBG response, it has not been yet fully investigated. We point out the merits of the approach, such as the functionality and simplicity of design, the accessibility and inexpensiveness of materials, the controllability and repeatability of processes, the drawbacks are also described, such as temperature effects, the problem of slipping of gages and the challenge of performing quasi-distributed strain measurements.

## 1. Introduction

Pipelines are naturally vulnerable to operational, environmental and man-made effects; there are many factors that could increase the vulnerability of pipelines: the mechanical deformation and ground movements caused by seismological effects; the leaks from neglect, vandalism or terrorism and the potential damages related to nearby excavations or illegal intrusions [1]. Pipeline monitoring and control actions are aimed at identifying and locating possible incipient or advanced failures, which are of enormous interest in the transport of hydrocarbons, but remain complex in application.

Buckling occurs in the presence of initial imperfections, which is typical in pipelines, particularly in submarine lines [2,3,4,5,6]. The loads commonly induced in pipelines are caused by the frictional restraint of axial extension due to temperature changes or internal pressure. Buckling can occur according to two different modes: the first involves an upward movement, the second provides snaking lateral movements. The lateral mode occurs at a lower axial load than the vertical one. Typical overall buckling occurs when the pipeline is located in trenches: initially, lateral constraints cause the pipeline to buckle upward and out of the trench; the second phase consists of the lateral and/or rolling movement of the part outside the trench [2].

In such a scenario, we focus on the application of optical fiber Bragg grating (FBG) sensors for the measurement of strain and bending along pipelines. Strain can be monitored by a series of FBG sensors installed on the outside of pipelines at discrete locations in the areas exposed to geohazard risks, such as landslides, earthquakes or settlements [1]. Particularly, we deal with the analysis of the theoretical background and laboratory experiments to check the reliability of the performance of this technology.

The popularity of optical fibers in real-time structural health monitoring has widely increased in the last decade. Both field applications and laboratory analysis have been widely conducted. Optical fiber sensors such as locally high-precision FBG and distributed Brillouin scattering time domain reflectometry (BOTDR) sensors are becoming very popular in many engineering applications because of their advantages with respect to conventional technologies, not only for their lower sensitivity to electromagnetic noise, but also as far as the accuracy, the durability and the sensing distance are concerned. The Bragg wavelength and the Brillouin frequency methods are suitable for measuring strain and temperature, as widely reported in the literature on the topic [7,8,9]. Experiments to monitor steel pipe and column buckling have been performed using a distributed Brillouin sensor system [10], to detect simultaneously both tension and compression: this suggests Brillouin sensors as a good tool for structural health monitoring. A combination of fiber Bragg and long-period gratings has been used to simultaneously measure strain and temperature with resolutions of ±9 με and ±1.5 °C over the ranges 290–1270 με and 25–50 °C [11]. Interferometric sensors such as Fabry–Perot are less amenable to multiplexing and forming a sensing network than FBGs [11]. So far, no relevant applications have been reported in the field of pipeline upheaval-buckling detection. In the oil and gas industry, the combination of FBG and BOTDR sensing technologies allows real-time monitoring and early warning systems; this approach is adopted to monitor the strain/stress state and safety of casing pipes in oil well operations [12]. The integration of these two monitoring systems could partially overcome the intrinsic weakness of each single technology, as FBGs are suitable for giving local information, meanwhile BOTDR is characterized by a series of limits such as poor spatial resolution, low test accuracy and low sampling frequency. The high cost of BOTDR systems for strain and temperature measurements is still a limit to the widespread use of FBG and BOTDR combinations for the real-time monitoring of pipelines. On the other hand, many studies about FBG sensors have mostly focused on the pipeline damage detection and leakage monitoring; only few studies deal with corrosion monitoring: in such a scenario, FBG hoop strain sensors look like a promising technology for monitoring pipeline corrosion and leakage [8].

We focus on the theoretical analysis of pipeline buckling and investigate the reliability of FBG monitoring both by examining the analytical model available and by performing a laboratory-scale experiment. The goal is to verify the extendibility (with appropriate adaptations and adjustments) of models and methods developed for the detection of pipeline upheaval buckling caused by externally imposed forces using Brillouin sensors to the case of Bragg gratings and service loads, in particular temperature. Although this factor is doubly important in view of its potentially disruptive effects on both pipelines (buckling) and FBGs (apparent thermal strain), it has not been yet fully investigated.

## 2. Materials and Methods

### 2.1. Theoretical Background

The problem essentially concerns a long straight uniform heavy elastic pipe, laid on a flat rigid base at ambient temperature in stress-free conditions, fixed to the base at both extremities and constrained to lie in the vertical plane [2,13,14]. If pressure and temperature are raised, a single symmetrical buckle will form. Obviously, the contour of the pipe in the uplifted configuration is greater than its original length, whence it follows that the axial force in the buckle must be less than that away therefrom (an effect known as “geometric shortening”).

**Figure 1:**
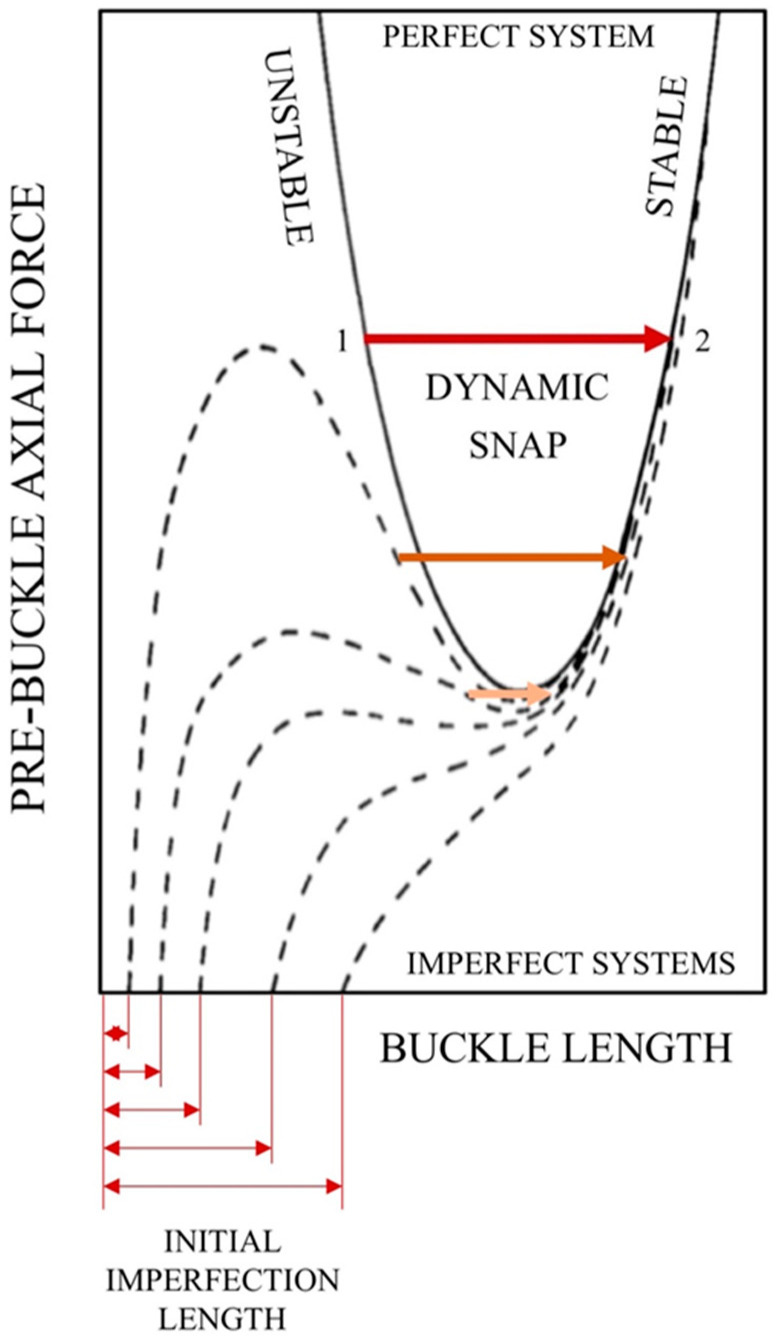
Imperfection sensitivity of equilibrium paths (adapted from Hobbs [2]): the transition from mechanical instability to stability happens at lower forces and less dramatically in the presence of an initial crookedness, to be eventually replaced by a progressive enlargement of the imperfection.

The curve of pre-buckle axial force (Figure 1) falls sharply for small lengths of the buckle and, after crossing a minimum, climbs steeply as the length grows. Such a shape implies that:

- A buckle cannot appear below a certain threshold of axial force;
- The equation of axial force admits two distinct solutions, associated to a shorter (always mechanically unstable) and a longer span respectively.

The transition from the unstable to the stable branch or “dynamic snap” happens at lower forces and less dramatically in the presence of an initial crookedness, to be eventually replaced by a progressive enlargement of the imperfection. Figure 1 sketches pre-buckle axial force (i.e., the axial force away from the buckle, which represents the control parameter of the phenomenon because it depends on the internal pressure and temperature rise)/buckle length curves for initially imperfect systems (dashed lines). Hobbs [2] demonstrated that “for very small imperfections a large snap is seen experimentally. As the initial out-of-straightness is increased, the snap occurs at lower forces and less dramatically. Eventually, for large enough imperfections the snap is eliminated, to be replaced by a single-valued magnification of the initial bow. For any initial imperfection the behavior is ultimately asymptotic to the stable curve for the perfect system”.

### 2.2. FBGs to Monitor Strain

A longitudinal sinusoidal variation in the refractive index of an optical-fiber core constitutes a fiber Bragg grating or FBG. It acts as an excellent band-stop filter in that virtually no backscatter occurs from each consecutive peak in the variation, except for wavelengths in the region of the “Bragg wavelength”. Local changes in strain or temperature linearly shift this parameter, thereby rendering the grating an intrinsic sensor [15,16,17]. The main technical specifications of FBGs are reported in Table 1, pointing out their advantages with respect to Brillouin-based sensors.

According to the basic structural-sensing equation for FBG strain gages, to obtain the mechanical strain, the measured one must be purged of the so-called “thermal output” (due to the discrepancy in the thermal coefficients of the host structure and the fiber), which can fatally vitiate measurements; furthermore, even if the thermal coefficients coincided, the fact that the sensitivity of FBGs to temperature is about an order of magnitude greater than that to strain could still pose serious difficulties [11].

### 2.3. Analytical Method

The measured longitudinal strain at an arbitrary point on the exterior of a buckled pipe equals the axial strain plus the maximum bending strain times the sine of the angle made by the point with the neutral axis of the pipe:

(1) $$\varepsilon_{l}=\varepsilon_{a}+\varepsilon_{b,\max}\sin\theta$$

**Figure 2:**
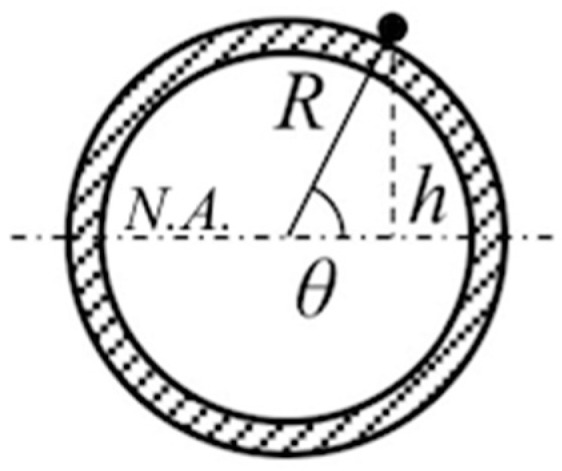
Schematic cross section of a pipe showing the main parameters of Equation (1): R, the outer radius; h, the distance from the neutral axis N.A.

This equation expresses the longitudinal strain ε_l_ as the sum of an axial ε_a_ (the pipe expands or contracts lengthways) and a bending ε_b_ (it also flexes because of buckling) component. The analytical model assimilates the pipe to an Eulerian strut; according to Navier’s formula, the bending strain equals the bending moment M times the distance h of the measuring point from the neutral axis N.A. of the pipe (Figure 2) divided by flexural rigidity or stiffness, product of the Young’s modulus E and the second moment of area I:

(2) $$\varepsilon_{b}=\frac{\mathrm{Mh}}{\mathrm{EI}}$$

The bending strain can be rewritten as the maximum bending strain ε_b,max_ (i.e., ε_b_ at θ = π/2 where h = R, the outer radius of the pipe) times sinθ:

(3) $$\varepsilon_{b}=\frac{\mathrm{MR}\sin\theta }{\mathrm{EI}}=\varepsilon_{b,\max}\sin\theta$$

Equation (1) contains three unknowns (namely the axial strain, the maximum bending strain and the angle) and can be solved if as many measurements of longitudinal strain per cross section are performed. The issue can be resolved as suggested by Feng et al. [18], who proposed that sensors should be arranged at intervals of 120° around the circumference (that is to say, at 12, 4 and 8 o’clock). A (determined) system of three equations in three unknowns must be solved:

(4) $$\varepsilon_{l,1}=\varepsilon_{a}+\varepsilon_{b,\max}\sin\theta$$

(5) $$\varepsilon_{l,2}=\varepsilon_{a}+\varepsilon_{b,\max}\sin\left(\theta +\frac{2\pi }{3}\right)$$

(6) $$\varepsilon_{l,3}=\varepsilon_{a}+\varepsilon_{b,\max}\sin\left(\theta +\frac{4\pi }{3}\right)$$

After some manipulation,

(7) $$\varepsilon_{a}=\frac{\varepsilon_{l,1}+\varepsilon_{l,2}+\varepsilon_{l,3}}{3}$$

(8) $$\theta =\tan^{-1}\left[\frac{2\varepsilon_{l,1}-\varepsilon_{l,2}-\varepsilon_{l,3}}{\sqrt{3}\left(\varepsilon_{l,2}-\varepsilon_{l,3}\right)}\right]$$

(9) $$\varepsilon_{b,\max}=\frac{\varepsilon_{l,2}-\varepsilon_{l,3}}{\sqrt{3}}$$

if θ = 0, π or else

(10) $$\varepsilon_{b,\max}=\frac{2\varepsilon_{l,1}-\varepsilon_{l,2}-\varepsilon_{l,3}}{3\sin\theta }$$

### 2.4. Experimental Set-Up

#### 2.4.1. Pipe

**Figure 3:**
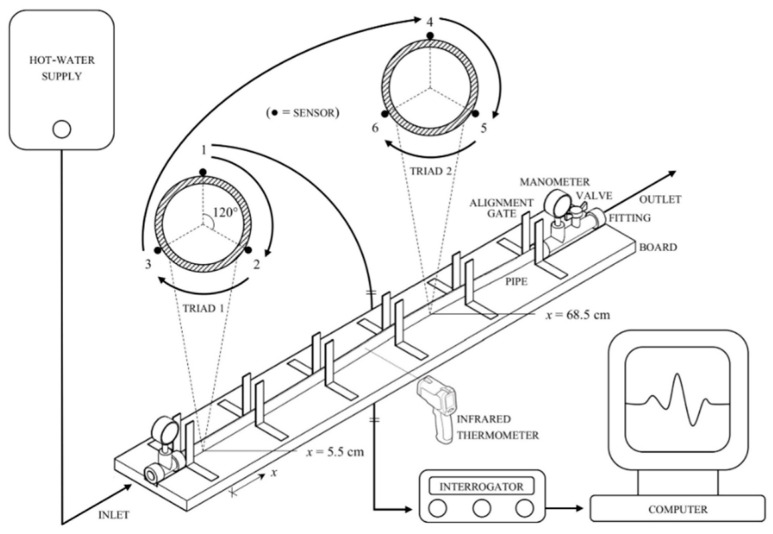
Diagram of the experiment: two threefold arrays of gages were fastened to a polyethylene tube connected to a supply of hot water, the internal pressure was attained by partially opening the outlet valve, temperature was measured with an infrared thermometer, the wavelength shift was acquired by an interrogator, transferred to a computer and converted to the longitudinal strain.

A 1-m-long LDPE PN-4 tube with an outer diameter of 20 mm, a wall thickness of 1.6 mm, a weight per unit length of 0.000934 N/mm, a yield strength of 14 N/mm^2^, a Young’s modulus of 200 N/mm^2^, a thermal coefficient of 200 με/°C, a Vicat softening temperature of 76–109 °C and a Poisson’s ratio of 0.45 was fitted with terminal blocks. Each block consisted of a brass tee, joined to a manometer at its upper end and to a 0.5-m-long polyethylene extension on the free side: one connected to a supply of hot water (an electric boiler), the other (including a ball valve) to a sink, both through a length of nylon-reinforced PVC hose, secured with steel worm-drive clamps. The resulting assembly was positioned on a spruce board with pantographically engraved recesses to receive the blocks, held in place by steel split-band clips, bolted to the plank. Two specular rows of six evenly spaced brass L-shaped brackets were screwed to the wooden base to prevent lateral movement of the tube with minimum friction (Figure 3).

#### 2.4.2. FBG Sensors and Interrogator

The optical sensing equipment (manufactured by Micron Optics) was comprised of epoxy-mountable FBG strain gages (model OS3100), working in the Bragg-wavelength range of 1532–1552 nm and characterized by a strain sensitivity of 10^−6^/με, a temperature sensitivity of 6.156 × 10^−6^ /°C, a thermal coefficient of 0.7 με/°C; an interrogator (model SI155), enabling hundreds of continuous measurements on four parallel channels with a sampling frequency of 1 kHz and an accuracy of 1 pm.

### 2.5. Procedure

Two threefold serial arrays of gages arranged in the manner described above (Section 2.3) were fastened with steel worm-drive clamps to the tube: the first (Triad 1) close to the inlet; the second (Triad 2) roughly at the center of a pre-existing bend, exploited to “catalyze” buckling (Section 2.1). The surface of the pipe was degreased with isopropanol, sandpapered and degreased again. After degreasing, gages were glued to the pipe with a superfast adhesive, resistant to high strain and temperature and specially designed for “difficult” plastics such as polyolefins. The steel worm-drive clamps, used to hold gages still during bonding, were left in place throughout the experiment to safeguard against detachment. In essence, a compromise was to be reached between the need for a material with sufficient flexibility and a relatively high thermal coefficient so as to obtain considerable expansions/deflections from small lengths, and effective fastening to a surface repelling most commercial fixatives. Subsequent inspection revealed no signs of break-off.

An internal pressure of 1 bar, as indicated by the dial of the manometers, was attained by partially opening the outlet valve; temperature was regularly measured with an infrared thermometer. The wavelength shift was acquired by the interrogator, transferred to a computer and converted to the longitudinal strain. The main technical specifications of the infrared thermometer are reported in Table 2.

**Figure 4:**
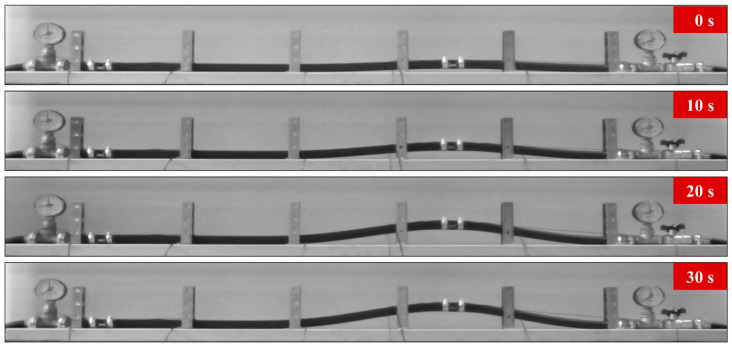
Selected frames from the video of the experiment (lasting 30 s): as the temperature was raised from 23.8 (room temperature) to 35.4 °C, buckling took place; a pre-existing bend in the tube was exploited to “catalyze” the process.

A video camera recorded the whole experiment: temperature was raised from 23.8 (room temperature) to 35.4 °C and buckling took place. A pre-existing bend in the tube was exploited to “catalyze” the process; this is evident in the 0-s frame in Figure 4, meanwhile the frames recorded at 10, 20 and 30 s reveal the magnitude of the buckling phenomenon due to temperature effects.

## 3. Results

The results are here presented by introducing the findings of the experimental activity, followed by the analysis of data modeling.

### 3.1. Experimental

**Figure 5:**
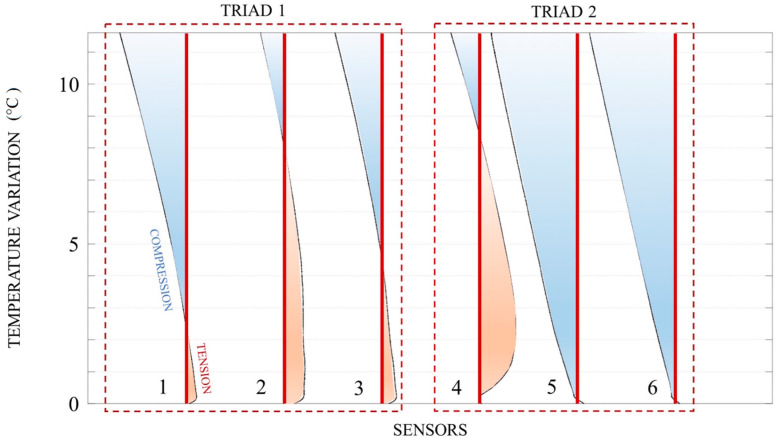
Temperature rise vs. longitudinal strain, where the longitudinal strain is placed on the horizontal axis; Triad 1 (sensors 1–3) registered an increment in tension (positive, highlighted in red), whereas Triad 2 recorded concurrent expansion in the upper portion of the tube (sensor 4) and contraction (negative, highlighted in blue) in the lower one (sensors 5–6).

As shown by the graph of the temperature rise vs. longitudinal strain (Figure 5), Triad 1 (sensors 1–3) registered an increment in tension (positive, highlighted in red), whereas Triad 2 recorded concurrent expansion in the upper portion of the tube (sensor 4) and contraction (negative, highlighted in blue) in the lower one (sensors 5–6), which means that the pipe bent, even if bending is not sufficient to deduce buckling, as explained in the next paragraph.

To facilitate their reading, data are presented as “wiggle traces” (i.e., lines oscillating about a null point: a common method of displaying seismic information, for instance). The traces ultimately become uninterpretable parallel lines on account of temperature compensation (Section 2.2), carried out by subtracting from the measured longitudinal strain the apparent thermal one, defined as:

(11) $${\Delta \varepsilon }_{T}\equiv \left(\alpha_{H}-\alpha_{F}+\frac{S_{T}}{S_{\varepsilon }}\right)\Delta T$$

where α denotes the thermal coefficients of the tube (H) and the fiber (F), S the fiber sensitivities to temperature (T) and strain (ε) and ΔT the temperature rise. During the experiment (lasting 30 s), temperature measurements were performed every 10 s; the following temperatures were recorded: at the beginning, 23.8 °C (room temperature); then 25.6 and 31.4 °C, and finally 35.4 °C at the end of the experiment. Temperature data were acquired with an accuracy of 0.1 °C. We interpolated the intermediate values using a polynomial of the third grade.

Figure 5 refers to the experimental results, produced by converting the acquired wavelength shift to the longitudinal strain and purging the latter of the apparent thermal strain. The basic structural-sensing equation for FBG strain gages was applied:

(12) $${\Delta \varepsilon }_{m}=\frac{1}{S_{\varepsilon }}\frac{{\Delta \lambda }_{B}}{\lambda_{B}}-{\Delta \varepsilon }_{T}$$

where Δε_m_ is the stress-related strain (exclusively mechanical), S_ε_ the strain sensitivity, Δλ_B_/λ_B_ the normalized Bragg-wavelength shift and Δε_T_ the apparent thermal strain. Step-by-step derivation of Equation (12) is given Appendix A.

Sensors 1–3 show different values of tension because a small upward concavity formed at the inlet (Figure 5), therefore the lower portion of the tube (sensors 2–3) stretched more than the upper one (sensor 1).

We got compression as the temperature change increased because the apparent thermal strain, enhanced by the relatively high thermal coefficient of the pipe material, far exceeded and in the end completely “masked” the mechanical one. As the experiment confirmed, the apparent thermal strain is the principal disadvantage of FBG sensors.

### 3.2. Modeling

Since its axial rigidity is generally several orders of magnitude greater than the flexural one, a thin-walled structure can absorb plenty of axial-strain energy without excessive distortion, whilst it must deform much more to take in an equivalent amount of bending-strain energy. If the strain energy is stored mostly as axial compression, it may be converted to bending during buckling. To bring about this transformation, significant deflections are needed [19]; as a consequence, the bending behavior is not necessarily indicative of buckling, being observable also in the phase preceding this event, which can nonetheless be unequivocally identified by distinctive trends of axial and bending strains, extracted from the measured longitudinal one:

- Condition 1, an increase in tensile strain or, correspondingly, a release of compression (geometric shortening – Section 2.1) within the bend;
- Condition 2, the generation of bending strain in the same tract [18].

The principle is analogous to that (familiar to all petroleum engineers) underlying the modern well-test analysis, whereby typical patterns of the pressure derivative (plotted vs. time on a bi-logarithmic graph) are individuated to “diagnose” flow regimes or geometries and, on the basis of their chronological sequence, the most suitable well/reservoir/boundary models is selected.

## 4. Discussion

**Figure 6:**
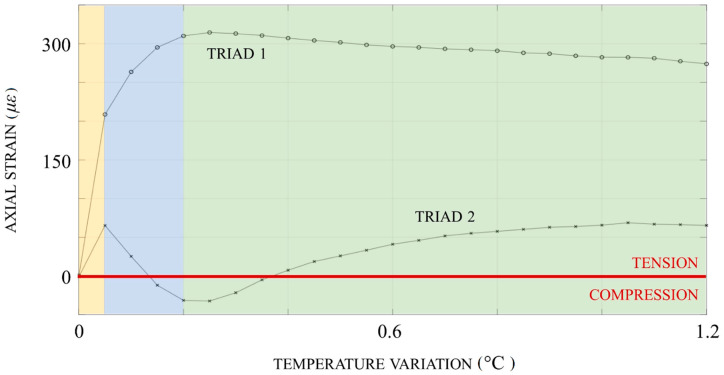
Axial strain vs. temperature rise: tension, encountered in the inceptive stage (yellow area), was gradually counterbalanced by the reaction of the constraint at the outlet, perceived first by Triad 2, undergoing sudden compression (blue area), which was subsequently released (green area).

The graph of axial strain vs. temperature rise (Figure 6) revealed that:

- The yellow area, tension (attributable to the instantaneous contact with the hot fluid) was encountered in the inceptive stage;
- The blue area, this was gradually counterbalanced by the reaction of the constraint at the outlet, perceived first by Triad 2, undergoing sudden compression;
- The green area, compression was subsequently released, a circumstance compatible with geometric shortening (Condition 1 was satisfied).

**Figure 7:**
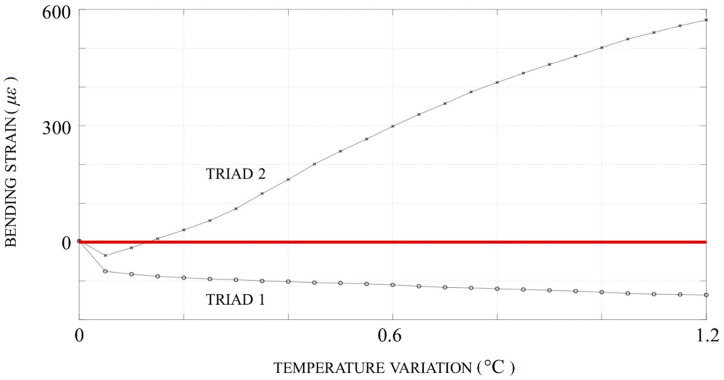
Bending strain vs. temperature rise: a prominent downward concavity (evidenced by a surge in bending strain) around the defect developed concomitantly with a much more modest upward concavity (negative) at the inlet.

The graph of the bending strain vs. temperature (Figure 7) evinced the concomitant development of:

- A prominent downward concavity (evidenced by a surge in bending strain: Condition 2 was fulfilled too) around the defect;
- A much more modest upward concavity (negative) at the inlet, as though the buckle “pushed against” the constraint and vice versa.

In the light of this evidence, we assessed that buckling was successfully detected. The longitudinal strain measured by an FBG sensor equals the inverse of the strain sensitivity times the normalized shift in the Bragg wavelength:

(13) $$\varepsilon_{l}=\frac{1}{S_{\varepsilon }}\frac{\Delta \lambda_{B}}{\lambda_{B}}$$

In the case under discussion (given the Bragg-wavelength range, interrogator accuracy and strain sensitivity mentioned in Section 2.4.2), the estimated uncertainty was less than 0.644–0.653 με; we assumed this to be a negligible quantity when compared with the orders of magnitude involved, reaching hundreds or thousands of microstrains.

Besides its intrinsic merits, such as the functionality and simplicity of design, the accessibility and inexpensiveness of materials, the controllability and repeatability of processes, the approach illustrated herein had some drawbacks worth pointing out:

- The low levels of applicable pressure did not permit a thorough evaluation of this factor;
- The temperature measurements, taken discontinuously on the external surface of the tube, required mathematical interpolation (Section 3.1) and were affected by heat dissipation;
- The gluing of gages to polyethylene was problematic and they might be prone to slip;
- Owing to the limited number of sensors, more relevant quasi-distributed strain measurements were not possible.

Despite the potential sources of inaccuracy just enumerated, the experimental results agree reasonably well with the analytical model and the observed physical phenomena. What emerged with absolute clarity was the severity of “temperature drift”, which remains a major obstacle to fruitful employment of optical sensing technology in this application field; however, innovative interrogation techniques such as that devised by Wu et al. [20] promise to overcome it.

It is difficult to draw a direct, exact comparison (at least quantitatively) between our results and those reported in the existing literature on the topic: to elucidate this, we shall now briefly review the most pertinent papers.

Ravet et al. [10] detected buckling in a steel pipe and column for the first time by means of distributed Brillouin fiber-optic sensors (DBFSs). The location of buckling was predetermined by thinning a small area of the specimen inner walls at mid-length. Each specimen was placed on a test bench and compressed stepwise, while deformation was constantly monitored by the sensors.

Feng et al. [18] worked out a method based on DBFSs for detecting the upheaval buckling of buried subsea pipelines. The model pipe was made of polyvinyl chloride (the preference for PVC over steel was dictated by the small scale of the experiment: the lower the stiffness, the easier it will be to trigger buckling); its initial shape was not straight due to imperfect manufacturing. The bottom of a trough was covered with a layer of compacted sand and a timber prop was put onto it to simulate the unevenness of the seabed. The pipe was accommodated inside the trough and, after the installation of sensors, completely buried. A hydraulic jack applied axial compression to one end of the pipe.

More recently, Feng et al. [21] combined the Brillouin optical time-domain analysis (BOTDA) with Raman optical time-domain reflectometry (ROTDR) to monitor strain and temperature respectively in a steel gas pipeline running under a busy Chinese road. The distributed temperature measurements enabled compensation for thermal effects and detection of leaks signaled by irregularities in the distribution.

All cited articles deal with distributed Brillouin sensors instead of Bragg-grating rosettes (Section 2.3) and with materials (steel, PVC) having mechanical properties that are very different from those of polyethylene (Section 2.4.1). Moreover, they focus on external forces (exerted by a test bench or a hydraulic jack) rather than service loads (e.g., the operating pressure and temperature—Section 2.5), apart from Feng et al. [21] where the pipe was subjected to an internal pressure of 16 bar and a temperature gradient of 9 °C. From a qualitative viewpoint, our observations are fairly consistent with those of Feng et al. [18], especially with regard to buckling “signatures” (Section 3.2).

Ling et al. [22] performed a similar study to check monitoring methods and design a circumferential strain measuring device. Their experimental study was conducted on a PVC model pipeline. Their results demonstrated good performance in the circumferential strain measurement, in agreement with our results. According to our encouraging results, a further investigation into FBG strain sensors applied to natural gas pipeline leakage detection will be performed; this is based on detecting negative pressure signals caused by a leakage. A similar approach has been suggested by Quingmin et al. [23]. They demonstrated that FBG strain sensors, used to monitor the hoop strain of a pipeline and detect negative pressure signals, are less influenced by noise than standard sensors.

## 5. Conclusions

The laboratory-scale experiment has been useful to check the reliability and drawbacks of FBG sensors for detecting the buckling of pipelines. The upscaling of the findings of the test is still challenging primarily because of the issues related to the low pressure and the temperature effects, which vary discontinuously on the external surface of the tube; this phenomenon depends on heat dissipation and requires some mathematical manipulation for the proper correction of the strain.

The positive aspects of using FBG sensors to monitor pipeline buckling, such as the reliability and repeatability of measurements, are in the real field hugely affected by the thermal issues, which remain a major obstacle to fruitful employment of optical sensing technology in this application field.

## Figures and Tables

**Table 1:** Main Technical Specifications of Fiber Bragg Gratings (FBGs) and Comparison with Brillouin-based Sensors [11].

Property	Sensor Type		Unit
	*Brillouin*	*Bragg*	
Strain sensitivity	4.6 × 10^−6^	1.0 × 10^−6^	/με
Temperature sensitivity	9.4 × 10^−5^	6.2 × 10^−6^	/°C
Spatial resolution	3–10	0.001	m
Temperature resolution	±1	±0.1	°C

**Table 2:** Main Technical Specifications of the Infrared Thermometer.

Temperature Range	−50–520 °C
Temperature Resolution	0.1 °C
Accuracy	±2%
Laser Wavelength	635–650 μm (Power < 1 mW)
Environmental Requirements	Temperature = 0–40 ° CHumidity ≤ 85%
Distance-to-spot Ratio	12:1

## Appendix A

Step-by-step derivation of Equation (12) is given here. The fundamental parameter of FBG sensors is the path length:

(A1) $$\zeta_{L}=n\Lambda$$

It is the product of the optical-fiber refractive index n and the grating period Λ, and a function of strain (or stress σ, in accordance with Hooke’s law) and temperature T:

(A2) $$\zeta_{L}=\zeta_{L}\left(\sigma ,T\right)$$

A 1st-order-truncated Taylor-series expansion of Equation (A2) gives:

(A3) $$\Delta \zeta_{L}={\left(\frac{\partial \zeta_{L}}{\partial \sigma }\right)}_{T}\Delta \sigma +{\left(\frac{\partial \zeta_{L}}{\partial T}\right)}_{\sigma }\Delta T$$

here (∂ζ_L_/∂σ)_T_ and (∂ζ_L_/∂T)_σ_ symbolize the derivatives of ζ_L_ with respect to σ and T at a reference state (σ_0_, T_0_). In the light of Equation (A1), Equation (A3) can be further expanded as:

(A4) $$\Delta \zeta_{L}=\left[n{\left(\frac{\partial \Lambda }{\partial \sigma }\right)}_{T}+\Lambda {\left(\frac{\partial n}{\partial \sigma }\right)}_{T}\right]\Delta \sigma +\left[n{\left(\frac{\partial \Lambda }{\partial T}\right)}_{\sigma }+\Lambda {\left(\frac{\partial n}{\partial T}\right)}_{\sigma }\right]\Delta T\Rightarrow$$

(A5) $$\Rightarrow \Delta \zeta_{L}=n\Lambda \left\{\left[{\left(\frac{\partial \varepsilon }{\partial \sigma }\right)}_{T}+\frac{1}{n}{\left(\frac{\partial n}{\partial \varepsilon }\right)}_{T}{\left(\frac{\partial \varepsilon }{\partial \sigma }\right)}_{T}\right]\Delta \sigma +\left[{\left(\frac{\partial \varepsilon }{\partial T}\right)}_{\sigma }+\frac{1}{n}{\left(\frac{\partial n}{\partial T}\right)}_{\sigma }\right]\Delta T\right\}$$

If the Young’s modulus E and the thermal coefficient α of the fiber are introduced, Equation (A5) becomes:

(A6) $$\Delta \zeta_{L}=n\Lambda \left\{\left[1+\frac{1}{n}{\left(\frac{\partial n}{\partial \varepsilon }\right)}_{T}\right]\frac{\Delta \sigma }{E_{F}}+\left[\alpha_{F}+\frac{1}{n}{\left(\frac{\partial n}{\partial T}\right)}_{\sigma }\right]\Delta T\right\}$$

the second addend in the first/second square bracket corresponding to the strain/thermo-optic effect. By defining the sensitivity to strain:

(A7) $$S_{\varepsilon }\equiv 1+\frac{1}{n}{\left(\frac{\partial n}{\partial \varepsilon }\right)}_{T}$$

and that to temperature:

(A8) $$S_{T}\equiv \alpha_{F}+\frac{1}{n}{\left(\frac{\partial n}{\partial T}\right)}_{\sigma }$$

Equation (A6) reduces to:

(A9) $$\frac{\Delta \zeta_{L}}{\zeta_{L}}=S_{\varepsilon }\Delta \varepsilon +S_{T}\Delta T$$

The normalized Δζ_L_ and Δλ_B_ (Bragg-wavelength shift) are proportional, so that:

(A10) $$\frac{\Delta \zeta_{L}}{\zeta_{L}}=\frac{\Delta \lambda_{B}}{\lambda_{B}}$$

In conclusion, the basic structural-sensing equation Equation (A9) takes the form:

(A11) $$\frac{\Delta \lambda_{B}}{\lambda_{B}}=S_{\varepsilon }\Delta \varepsilon +S_{T}\Delta T$$

and suggests that any Δλ_B_ caused by a deviation in temperature is indistinguishable from one ascribable to the application of an exclusively mechanical force; therefore, some sort of compensation is needed. Imagine that a fiber is installed on a host structure not subjected to external loads but experiencing only a ΔT; Then an axial stress will develop therein, owing to the discrepancy in α:

(A12) $$\Delta \sigma =E_{F}\left(\alpha_{H}-\alpha_{F}\right)\Delta T$$

Substitution of Equation (A12) into Equation (A11) yields:

(A13) $$\frac{\Delta \lambda_{B}}{\lambda_{B}}=\left[S_{\varepsilon }\left(\alpha_{H}-\alpha_{F}\right)+S_{T}\right]\Delta T$$

and, allowing for stress-related strain:

(A14) $$\frac{\Delta \lambda_{B}}{\lambda_{B}}=S_{\varepsilon }\left[\Delta \varepsilon_{m}+\left(\alpha_{H}-\alpha_{F}\right)\Delta T\right]+S_{T}\Delta T$$

Rearrangement of Equation (A14) delivers:

(A15) $$\Delta \varepsilon_{m}=\frac{1}{S_{\varepsilon }}\frac{\Delta \lambda_{B}}{\lambda_{B}}-\Delta \varepsilon_{T}$$

in which:

(A16) $$\Delta \varepsilon_{T}\equiv \left(\alpha_{H}-\alpha_{F}+\frac{S_{T}}{S_{\varepsilon }}\right)\Delta T$$

stands for the apparent thermal strain.
